# Sociodemographic Correlates of Human Papillomavirus Vaccine Uptake: Opportunistic and Catch-Up Vaccination in Norway

**DOI:** 10.3390/cancers13143483

**Published:** 2021-07-12

**Authors:** Li Dong, Mari Nygård, Bo T. Hansen

**Affiliations:** 1 Department of Research, Cancer Registry of Norway, 0304 Oslo, Norway; dongli@sxu.edu.cn (L.D.); Mari.Nygard@kreftregisteret.no (M.N.); 2 Institutes of Biomedical Sciences, Shanxi University, Taiyuan 030006, China

**Keywords:** human papillomavirus, opportunistic vaccination, catch-up vaccination, sociodemographic, inequalities, cervical cancer

## Abstract

**Simple Summary:**

HPV vaccination protects against virus that may cause cervical cancer. Opportunistic HPV vaccination (i.e., vaccination at a citizens’ own initiative and cost) has been available in Norway since the first HPV vaccine was licensed in 2006. A routine HPV vaccination program targeting 12-year-old girls was introduced in 2009. A delayed catch-up vaccination program was initiated in 2016, offering HPV vaccination free-of-charge to women born in 1991 and later who had not previously been vaccinated in the routine program. The aim of this study was to assess sociodemographic correlates of opportunistic and catch-up HPV vaccine uptake among women in Norway. We found inequalities in both self-paid opportunistic and free-of-charge catch-up HPV vaccine uptake among adolescents and adult women, with particularly low uptake among women with two immigrant parents and among women with a low household income.

**Abstract:**

Achieving equity in human papillomavirus (HPV) vaccination has high priority. In this nationwide registry-based study, we aimed to investigate sociodemographic correlates of HPV vaccine uptake among women who were vaccinated opportunistically at their own initiative and cost during October 2006–June 2018, and among women who were vaccinated free-of-charge in a catch-up vaccination program during November 2016–June 2018. For 840,328 female residents born in Norway between 1975 and 1996, we retrieved HPV vaccination and sociodemographic data from national registries. We used separate models to analyze the sociodemographic correlates of the initiation and completion of HPV vaccination in opportunistic and catch-up vaccination settings. Overall initiation rate for opportunistic HPV vaccination was 2.2%. Uptake increased consistently with birth year, maternal education level, and household income. Having two immigrant parents or a mother working in a lower prestige occupation was strongly associated with low opportunistic vaccination uptake. Similar but weaker inequities were observed in catch-up HPV vaccination. Initiation rate during the first 20 months of the catch-up program was 46.2%. Completion rate was 72.1% and 73.0% for opportunistic or catch-up vaccination, respectively, with small inequities. In conclusion, HPV vaccine uptake was strongly associated with sociodemographic background both in opportunistic and catch-up vaccination settings, with particularly low uptake associated with having two immigrant parents and low household income.

## 1. Introduction

Human papillomavirus (HPV) has a predilection for infecting cutaneous and mucosal epithelial cells and subsequently causes almost all cervical cancer and a substantial fraction of other anogenital and oropharyngeal cancers [1,2]. It is estimated that on average, 4.5% of all cancers worldwide are attributable to HPV [3], in addition to non-malignant conditions such as genital warts and juvenile onset of recurrent respiratory papillomatosis [4]. Therefore, vaccination against HPV has a high potential to improve public health. 

HPV vaccines were developed against different HPV types, with a bivalent (2v) vaccine providing protection against HPV16, 18, a quadrivalent (4v) vaccine against HPV6, 11, 16, 18 and a nonavalent (9v) vaccine against HPV6, 11, 16, 18, 31, 33, 45, 52, 58. All HPV vaccines have proven to be safe, highly immunogenic, and effective against vaccine-type HPV infection and high-grade cervical lesions in clinical trials [5]. The HPV vaccines are prophylactic, and thus most effective if given to HPV-naïve individuals. However, catch-up vaccination of older birth cohorts may be cost-effective, although the value for money generally decreases with increasing age at vaccination and the upper age limit for a cost-effective intervention varies greatly between studies, depending on setting, model type, and underlying assumptions [6].

Countries that have achieved high vaccination coverage have also observed 73–85% decline in vaccine-type HPV prevalence, and declines of 41–57% in high-grade cervical lesions among young women less than 10 years after implementation of HPV vaccination [7]. A total of 90% HPV vaccination coverage in the targeted population is key to achieve the recently established ambitious goal to eliminate cervical cancer by the World Health Organization [8]. Herd effects may also offer protection in populations with high vaccination coverage [9]. However, HPV vaccination coverage varies by region and income level worldwide [10]. A recent review of HPV vaccination coverage, policies, and practical implementation across 31 European countries showed that school delivery within structured HPV vaccination programs and use of reminders favored high coverage [11]. 

Sociodemographic factors such as education level, socioeconomic status, beliefs, and immigration status may be associated with attitudes toward general health, vaccination in general as well as HPV vaccination, and thereby HPV vaccine uptake [12,13]. Uptake inequities have been documented in routine school delivery and catch-up HPV vaccination settings, even when vaccination is free-of-charge or subsidized [13,14,15,16,17,18]. Fewer studies have addressed potential inequities in a self-paid opportunistic vaccination setting, which still predominates in some countries, particularly those with low- and middle-income levels that have a relatively high cervical cancer burden [10]. Given that equity in access to health care is a priority [19], knowledge on the social profile of HPV vaccine uptake is needed to assess to what extent equity is achieved, in order to inform decision-making in health care. 

The first HPV vaccine was licensed in Norway in October 2006, and self-paid opportunistic vaccination with a recommended three-dose schedule has since been available. Opportunistic vaccination happens at a citizen’s request to a physician and is thus not part of a vaccination program. In 2009, free of charge HPV vaccination became part of the Norwegian childhood vaccination program, starting off with routine HPV vaccination administered at school to girls in the 7th grade [16]. Regular tenders are issued to decide which HPV vaccine is offered in the program. The 4v vaccine in a three-dose schedule was offered to girls in the routine program during 2009–2017 (birth cohorts 1997–2004), while the 2v vaccine in a two-dose schedule was offered from 2017 to date (birth cohorts 2005 and later). In 2018, boys in the 7th grade (birth cohorts 2006 and later) were included in the routine HPV vaccination program, and they were offered the 2v vaccine in a two-dose schedule. Catch-up vaccination was not initiated simultaneously with the routine programs in Norway, but a delayed catch-up vaccination program with the 2v vaccine in a three-dose schedule was effective from November 2016 through to June 2019, targeting women born in 1991 and later who had not been previously vaccinated in the routine program. Catch-up vaccination was offered free-of-charge through primary care services.

We performed a nationwide registry-based study to investigate sociodemographic correlates of HPV vaccination in self-paid opportunistic and free-of-charge catch-up vaccination settings among women in Norway, aiming to identify barriers to achieving the targets set by the WHO’s global strategy to accelerate the elimination of cervical cancer as a public health problem.

## 2. Materials and Methods

### 2.1. Study Population and Data Collection

**Figure 1:**
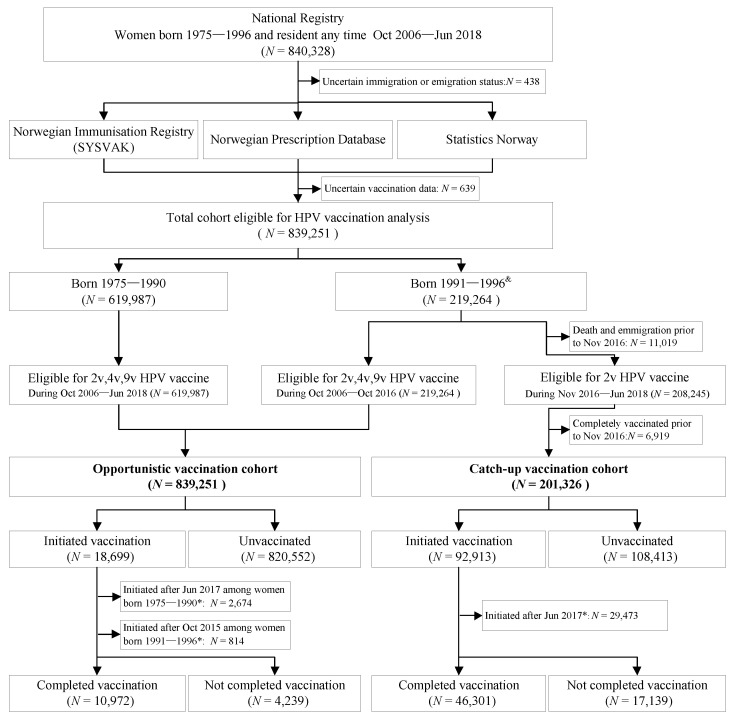
Data sources and study population for opportunistic and catch-up HPV vaccination. Abbreviations: PIN, personal identification number; HPV, human papillomavirus. ^&^ Women born from 1991 to 1996 were eligible for opportunistic vaccination until October 2016 and for the 2v catch-up vaccination program from November 2016; * Women who initiated after one year before the end of follow-up were excluded from completion analyses because their completion during one year could not be assessed.

All data in this population-based study were extracted from nationwide registries, which were linked on an individual level via the unique personal identification number (PIN) that is given to each Norwegian citizen at birth or immigration (Figure 1). We focused on women because HPV vaccination among men in Norway was negligible during the follow-up period of the present study. We identified all women in the National Registry who were born between 1975 and 1996 and were resident in Norway at any time during October 2006 to June 2018, and extracted their dates of birth, death, immigration and emigration as well as their region of residence.

From Statistics Norway, we extracted information for each cohort member on parental country of birth, maternal education level, maternal occupation, and household income after tax. We used socioeconomic data from the calendar year when the subject became eligible for HPV vaccination, which was defined as the last occurrence among the year of the ninth birthday (the youngest recommended age for HPV vaccination in Norway), 2006 (the year for licensure of the first HPV vaccine), or the year of immigration to Norway.

Information on HPV vaccination for each cohort member was obtained for the period October 2006–June 2018. We extracted dates of vaccination and prescription for each dose from the Norwegian Immunization Registry (SYSVAK) and the Prescription Registry, respectively, for all HPV vaccines available in Norway during this period. We used data from SYSVAK when available, and supplemented with data from the Prescription Registry if women were registered with less than three doses of the same vaccine type in SYSVAK and there were additional data from the Prescription Registry.

### 2.2. Opportunistic and Catch-Up Vaccination Cohorts: Definitions and Follow-Up

We defined opportunistic vaccination as any vaccination happening outside the organized routine or catch-up program. Opportunistically vaccinated women have to pay for the vaccination themselves, which currently has a cost of €300–400 for three doses (i.e., complete vaccination as recommended). Prices have been similarly high throughout the study period, although it varies some by calendar year and vaccine type. The entire study cohort was eligible for opportunistic HPV vaccination. Women born from 1975 to 1990 were not age-eligible for catch-up vaccination and any HPV vaccination administered during the follow-up period of October 2006–June 2018 was considered opportunistic for these birth cohorts. Women born from 1991 to 1996 contributed to the opportunistic vaccination cohort until the start of the catch-up program (i.e., until October 2016), as seen in Figure 1. The catch-up vaccination cohort included women born from 1991 to 1996 who were living in Norway while they were eligible for catch-up vaccination and who had not been fully vaccinated opportunistically before the start of the catch-up program in November 2016. This cohort was followed-up for 2v vaccination for the period of November 2016–June 2018 (Figure 1). None of the women included in this study were eligible for routine school-based HPV vaccination.

Women who did not receive any dose of HPV vaccine during follow-up were classified as unvaccinated. Women eligible for opportunistic vaccination who received at least one dose of any HPV vaccine were classified as having initiated opportunistic vaccination, and women eligible for catch-up vaccination who received at least one dose of the 2v vaccine were classified as having initiated catch-up vaccination. Women who received at least three doses of the same HPV vaccine within one year were classified as having completed HPV vaccination (Figure 1). Completion rates were calculated for women who had at least one year of follow-up time after initiation.

### 2.3. Statistical Analysis

Opportunistic and catch-up vaccination were assessed in separate models. We used Cox regression models and reported hazard ratios (HR) and 95% confidence intervals (CI) in the analyses of HPV vaccination initiation. We used logistic regression models and reported odds ratios (ORs) and 95% CI in the analyses of HPV vaccination completion among women who initiated HPV vaccination. We performed unadjusted univariate analyses of each independent variable as well as adjusted multivariate analyses with several of the independent variables included in the same model. The same variables with the same categories were addressed for all outcomes. For ordinal variables, we chose the lowest category as the reference level, while for nominal variables, we generally chose the category with the highest number of women as the reference category. Individuals with missing values for a sociodemographic variable were excluded from models including this variable, thus sample size differed between models. Due to the high proportion of missing values for maternal education and maternal occupation resulting from relatively low completeness for these variables at Statistics Norway, we did not include these variables in the adjusted models. Statistical analysis was performed with R Studio version 3.6.1. All tests were two-sided, and *p*-values less than 0.05 were considered statistically significant.

## 3. Results

A total of 840,328 women born in the period 1975–1996 and resident in Norway at any time during the period October 2006 to June 2018 were identified. We excluded 438 women with uncertain immigration/emigration status and 639 women with uncertain vaccination data, resulting in a cohort of 839,251 women eligible for analyses (Figure 1).

### 3.1. Overall HPV Vaccination Initiation and Completion Rate

Among the 839,251 women eligible for opportunistic HPV vaccination, 1476, 16,976 and 929 women had at least one dose of the 2v, 4v, or 9v vaccine, respectively, of which a total of 18,699 women had at least one dose of any kind of vaccine. The initiation rate was thus 2.2% in the opportunistic setting. Among the 201,326 women eligible for catch-up vaccination during the study period, 92,913 had at least one dose of the 2v vaccine, resulting in an initiation rate of 46.2% during the first 20 months of the catch-up program (the program continued after the end of follow-up of the present study). The median age at initiation of HPV vaccination was 21.3 years (interquartile range: 16.4–27.6 years) in the opportunistic setting, and 23.3 years (interquartile range: 21.8–24.8 years) in the catch-up setting. HPV vaccination completion rate among women who had initiated vaccination was 72.1% and 73.0% for opportunistic and catch-up HPV vaccination, respectively.

### 3.2. Sociodemographic Characteristics Associated with Initiation and Completion of Opportunistic HPV Vaccination

There were considerable differences in the initiation rate of opportunistic HPV vaccination for most of the sociodemographic variables (Table 1). For year of birth, a progressive increase in the initiation rate was observed from 0.4% in the oldest birth cohort to 5.5% in the youngest birth cohort (adjusted HR: 21.25, 95% CI: 19.45–23.21, *p* < 0.001). The initiation rate also differed significantly by parental country of birth. Women with two immigrant parents were far less likely to initiate opportunistic HPV vaccination compared to women with two Norwegian-born parents, with initiation rates of 0.6% and 2.9%, respectively (adjusted HR: 0.58, 95% CI: 0.55–0.62, *p* < 0.001). However, women with one foreign-born and one Norwegian-born parent had the highest uptake at 3.8% (adjusted HR: 1.17, 95% CI: 1.11–1.23, *p* < 0.001).

Maternal education level showed a strong and consistently positive association with opportunistic HPV vaccination initiation. The opportunistic initiation rates increased from 1.6% among women whose mothers had the lowest education to 8.3% among women whose mothers had the highest education (unadjusted HR: 5.79, 95% CI: 5.46–6.14, *p* < 0.001). Maternal occupation was also associated with opportunistic HPV vaccine initiation. Compared to women with mothers in higher prestige occupations, women with mothers in lower prestige occupations had a lower opportunistic initiation rate (unadjusted HR: 0.44, 95% CI: 0.42–0.45, *p* < 0.001). A consistently positive association was observed for household income, with the initiation rate increasing from 1.3% in the lowest income bracket to 6.9% in the highest income bracket (adjusted HR: 2.51, 95% CI: 2.36–2.67, *p* < 0.001). There were small but significant differences in opportunistic HPV vaccine uptake between geographical regions, ranging from 1.8% in Mid-Norway to 2.8% in West-Norway (Table 1).

The analyses addressing completion of opportunistic HPV vaccination (Table 2) showed similar patterns to those reported for initiation of opportunistic HPV vaccination, although the relative differences between the categories of each variable were often smaller or non-significant in the completion analyses, especially in the adjusted model. The lowest completion rates of opportunistic vaccination were observed in the oldest birth cohort (56.5%), among women with two immigrant parents (65.2%), and among women in the lowest income bracket (67.1%). Year of birth was most strongly associated with the completion of opportunistic HPV vaccination, with the youngest women having a higher rate of completion than the oldest women (adjusted OR: 2.93, 95% CI: 2.34–3.67, *p* < 0.001). Women with two immigrant parents also had a lower likelihood of completing opportunistic vaccination compared to women with two Norwegian-born parents (adjusted OR: 0.77, 95% CI: 0.67–0.87, *p* < 0.001). Unlike the corresponding initiation analyses, a progressively positive association was not observed between opportunistic vaccination completion and maternal education or household income, and there was no association with maternal occupation.

### 3.3. Sociodemographic Characteristics Associated with Initiation and Completion of Catch-Up HPV Vaccination

The overall patterns between sociodemographic status and the initiation of catch-up HPV vaccination (Table 3) were similar to those observed for opportunistic vaccination (Table 1). A strikingly low catch-up initiation rate (21.1%) was observed among women with two immigrant parents compared to 53.9% among women with two Norwegian-born parents (adjusted HR: 0.40, 95% CI: 0.39–0.41, *p* < 0.001). A total of 45.1% of women with one Norwegian-born and one foreign-born parent initiated catch-up vaccination compared to 53.9% of those with two Norwegian-born parents (adjusted HR: 0.82, 95% CI: 0.80–0.84, *p* < 0.001). Women with mothers with the lowest education level had a relatively low catch-up initiation rate (40.4%), especially when compared to women with the most highly educated mothers (57.9%; unadjusted HR: 1.64, 95% CI: 1.59–1.69, *p* < 0.001). The highest catch-up initiation rate was observed among women with mothers in higher prestige occupations (59.5%), which was nearly 10 percentage points higher than among women with mothers working in lower prestige occupations (49.6%, unadjusted HR: 0.76, 95% CI: 0.75–0.78, *p* < 0.001). Women in households with the lowest income had a very low catch-up initiation rate (29.0%) in contrast to women in households with the highest income (54.7%; adjusted HR: 1.60, 95% CI: 1.54–1.66, *p* < 0.001).

Among women who initiated catch-up HPV vaccination, there was little difference in completion across the categories of each variable. However, most patterns observed for the initiation of catch-up vaccination were also observed for completion, although the absolute and relative effect sizes were smaller (Table 4).

## 4. Discussion

This nationwide population-based cohort study examined sociodemographic status in relation to the initiation and completion of opportunistic and catch-up HPV vaccination in Norway. We found a very low initiation rate (2.2%) and a suboptimal completion rate (72.1%) of opportunistic HPV vaccination between 2006 and 2018 among Norwegian women born 1975–1996. The catch-up program was not yet completed by the end of follow-up of the present study, hence the final uptake rate for the entire catch-up program is higher than reported here. Several sociodemographic characteristics were strongly associated with uptake of opportunistic HPV vaccination with the lowest initiation and completion rates observed among women in older birth cohorts, women with two immigrant parents, women with mothers with the lowest education, and women living in households with the lowest income. A similar pattern was observed for HPV catch-up vaccination, a setting with a much higher uptake rate.

We observed that women in younger birth cohorts were more likely to initiate and complete vaccination, particularly in the opportunistic setting. HPV vaccination at a young age is important because HPV vaccine efficacy is low among those previously exposed to HPV infection [20,21,22]. The median age at first intercourse among women in Norway is 17 years [23], whereas the median age of opportunistic and catch-up HPV vaccination in the present study was 21–23 years. The median number of sexual partners among Norwegian women aged 18–24 is estimated to be five [23]; thus, most Norwegian women vaccinated against HPV opportunistically or in the catch-up program are likely to have had multiple sexual partners and to have been exposed to HPV before they were vaccinated [24]. Compared to the routine vaccination of pre-adolescent girls, a lower effectiveness of opportunistic and catch-up vaccination is thus to be expected in Norway. Similarly, extending the catch-up program to even older birth cohorts is also expected to yield a relatively low effectiveness since the incremental benefit of vaccinating older women decreases with age. However, the upper age limit for a cost-effective catch-up intervention varies between studies and depends on a range of conditions [6,25,26,27] and the value of providing additional catch-up vaccination to women born before 1991, who thus far have not been eligible for routine or catch-up vaccination in Norway, remains uncertain.

Completion of the vaccination series was suboptimal in both vaccination settings investigated, which is likely to further reduce the effectiveness of HPV vaccination in the cohorts studied here. Raised awareness toward completing the vaccine series among adolescent and adult women seems warranted. Opportunistic and catch-up vaccination in Norway requires attendance at a general practitioner, pharmacy, or a public health clinic, which in part may explain why the completion rate is lower than in the routine school-based setting [16] where vaccination is probably somewhat more easily accessible. In the opportunistic setting, the additional cost of the second and third dose may also deter completion.

Parental country of birth was one of the strongest predictors of both initiation and completion of opportunistic and catch-up HPV vaccination. We found that women with two immigrant parents were far less likely to initiate HPV vaccination compared to women with two Norwegian-born parents. These results are in line with findings of subsidized opportunistic vaccination in Sweden [28] and catch-up HPV vaccination status in England and Sweden [28,29,30]. Cultural norms, ethnicity, and religious beliefs may be associated with health behavior and HPV vaccination status [30,31]. A recent systematic review revealed that access to information, concerns about vaccine safety and promiscuity, providers’ recommendations, school mandates, financial issues, immigration laws, and living in disadvantaged neighborhoods were factors that may influence HPV vaccination uptake among girls/women with immigrant parents [32]. The low HPV vaccine uptake documented here among women with immigrant parents suggests that special efforts are needed to reach this underserved part of the population to make vaccination more equitable.

Household income was strongly correlated with HPV vaccine uptake in both vaccination settings. Financial status has also been shown to be important for opportunistic HPV vaccine uptake in China [33], and Sweden [28,34] as well as in catch-up programs in Sweden [28], Australia [35], and the Netherlands [36]. We also observed that there was a consistent gradual increase of HPV vaccine uptake with each increasing income level in Norway. Moreover, the relative differences were somewhat larger in the opportunistic than in the catch-up setting, which may be associated with the corresponding difference in the cost of vaccination. A previous study observed smaller concordant uptake differences in the routine free-of-charge school-based vaccination setting [16], indicating that this is the least inequitable HPV vaccination setting, at least in Norway.

We noted some regional differences in both opportunistic and catch-up HPV vaccine uptake. We do not know what caused these differences, but one possibility is vaccine accessibility. The regions differ in population density, which could impact on the effort and cost of reaching a vaccine provider. Moreover, vaccine delivery in the childhood vaccination program is administered by local municipality health services [37] and the execution of the temporary catch-up program may not have been identical throughout the country even though the guidelines were national. Our observations regarding regional uptake differences are echoed in the statistics from the catch-up program, which lasted another year after the end of the follow-up of the present study. It reached a national uptake level of approximately 60%, which varied from 52% in some counties in the eastern region to 66% in some counties in the middle and western regions [38].

Previous studies on the associations between sociodemographic characteristics and HPV vaccine uptake have mainly focused on uptake among children and young adults [13,36,39]. The present study found that maternal and household characteristics were also relevant among adult women. For women who were financially dependent on their parents, the high out-of-pocket cost of opportunistic HPV vaccination could be directly associated with the observed inequalities relating to parental income. However, the inequalities observed in the free-of-charge catch-up program indicates that a broader family context may also be important for the preventive health care decisions of adults. Factors such as family awareness and attitudes related to preventive health care or HPV may be of importance for the decision to have the HPV vaccine or not, and may also be associated with the inequalities observed here. Such factors may contribute directly if the family to some extent governs the woman’s decision, or indirectly through social inheritance.

Several of the sociodemographic variables included in the present study are correlated with each other, as indicated by the observation that estimates of the same variable sometimes differed between unadjusted and adjusted models. However, most effects observed in the unadjusted models were also significant after adjustment, indicating that the variables also contributed independently to the association with HPV vaccine uptake.

All data used in this study were extracted from nationwide registers, which ensures objective data and avoids selection and response bias. Moreover, this study had a large sample size and high power to detect differences in HPV vaccine uptake. However, the analyses presented here do not address causal relationships and cannot identify the factors that motivate the decision whether or not to have the HPV vaccine. Moreover, although we presented adjusted analyses, residual confounding is likely. Further limitations of this study are the high proportion of missing data for maternal education and occupation, which limits the inference that can be drawn regarding these variables, and the fact that we do not have access to data of HPV vaccination that may have happened outside Norway. Finally, the catch-up program was not entirely completed by the end of follow-up of the present study, thus the catch-up results presented do not constitute the entire catch-up program.

## 5. Conclusions

Self-paid opportunistic HPV vaccination among Norwegian women is characterized by a very low initiation rate, a suboptimal completion rate, and a high age at vaccination, with the lowest uptake among women of low socioeconomic status and women with two immigrant parents. Thus, the opportunistic HPV vaccination setting is inefficient from a public health perspective and does not promote equity in access to preventive health care. Providing free-of-charge vaccination to women in their 20s through a catch-up program greatly increases the initiation rate compared to opportunistic vaccination, but delayed catch-up vaccination also suffers from inequalities and suboptimal completion. Targeted campaigns in catch-up vaccination programs toward immigrants and socially deprived groups could mitigate the disparities observed in the present study.

## Figures and Tables

**Table 1:** Sociodemographic characteristics of HPV vaccination initiation among women eligible for opportunistic HPV vaccination in Norway.

Characteristics	No. Total	No. Initiated	% Initiated	Unadjusted HR (95% CI)	Adjusted HR (95% CI) ^§^
Overall	839,251	18,699	2.2	--	--
***Year of birth***					
1975–1978	143,328	573	0.4	1 (reference)	1 (reference)
1979–1981	112,280	915	0.8	2.16 (1.95, 2.40) ***	2.24 (2.02, 2.49) ***
1982–1984	116,404	1392	1.2	3.34 (3.03, 3.68) ***	3.47 (3.15, 3.83) ***
1985–1987	121,389	1947	1.6	4.56 (4.16, 5.01) ***	4.45 (4.05, 4.89) ***
1988–1990	126,586	4502	3.6	9.84 (9.02, 10.73) ***	8.87 (8.13, 9.69) ***
1991–1993	114,834	3647	3.2	14.55 (13.3, 15.92) ***	13.04 (11.91, 14.28) ***
1994–1996	104,430	5723	5.5	23.99 (21.97, 26.18) ***	21.25 (19.45, 23.21) ***
***Parental country of birth***					
Two Norwegian-born parents	533,302	15,642	2.9	1 (reference)	1 (reference)
Two immigrant parents	248,458	1509	0.6	0.35 (0.33, 0.37) ***	0.58 (0.55, 0.62) ***
One foreign-born and one Norwegian-born parent	40,631	1531	3.8	1.37 (1.30, 1.45) ***	1.17 (1.11, 1.23) ***
***Region of residence***					
East	397,929	9456	2.4	1 (reference)	1 (reference)
South	45,022	931	2.1	0.82 (0.77, 0.88) ***	0.77 (0.72, 0.83) ***
West	175,095	4840	2.8	1.14 (1.11, 1.18) ***	1.05 (1.01, 1.08) ***
Middle	108,270	1931	1.8	0.72 (0.68, 0.75) ***	0.68 (0.64, 0.71) ***
North	75,326	1493	2.0	0.80 (0.76, 0.84) ***	0.74 (0.70, 0.78) ***
***Maternal education***					
None/Primary only/Lower secondary	160,967	2501	1.6	1 (reference)	--
Upper secondary/Post-secondary non-tertiary	266,859	6152	2.3	1.48 (1.41, 1.55) ***	--
Undergraduate	161,416	7329	4.5	3.02 (2.89, 3.16) ***	--
Postgraduate	23,830	1967	8.3	5.79 (5.46, 6.14) ***	--
***Maternal occupation***					
Managers/Professionals/Technicians	192,577	9011	4.7	1 (reference)	--
Others ^#^	220,633	4615	2.1	0.44 (0.42, 0.45) ***	--
***Household annual income after tax (NOK)***					
<289,448	262,025	3388	1.3	1 (reference)	1 (reference)
289,448–452,141	188,441	2859	1.5	1.12 (1.07, 1.18) ***	0.81 (0.77, 0.85) ***
452,141–603,667	190,584	4424	2.3	1.65 (1.58, 1.72) ***	0.97 (0.92, 1.02)
603,667–797,912	114,761	4364	3.8	2.70 (2.58, 2.83) ***	1.35 (1.29, 1.42) ***
797,912–977,605	35,231	1875	5.3	3.85 (3.63, 4.07) ***	1.90 (1.79, 2.02) ***
977,605–	25,775	1770	6.9	5.26 (4.96, 5.57) ***	2.51 (2.36, 2.67) ***

§ Estimates adjusted for all variables in the table (N = 839,251) except for maternal education level and maternal occupation, which were not included due to a high proportion of missing values. ^#^ Others refers to clerical support workers, service and sales workers, skilled agricultural, forestry and fishery workers, craft and related trades workers, plant and machine operators and assemblers and elementary occupations. *** *p* < 0.0001. Abbreviations: HR: hazard ratios; CI: confidence interval.

**Table 2:** Sociodemographic characteristics of HPV vaccination completion among women who initiated opportunistic HPV vaccination in Norway.

Characteristics	No. Initiated	No. Completed	% Completed	Unadjusted OR (95% CI)	Adjusted OR (95% CI) ^§^
Overall	15,211	10,972	72.1	--	--
***Year of birth***					
1975–1978	347	196	56.5	1 (reference)	1 (reference)
1979–1981	675	445	65.9	1.49 (1.14, 1.94) **	1.51 (1.16, 1.97) **
1982–1984	1048	730	69.7	1.77 (1.38, 2.27) ***	1.79 (1.39, 2.31) ***
1985–1987	1394	872	62.6	1.29 (1.01, 1.63) *	1.33 (1.04, 1.69) *
1988–1990	3191	2063	64.7	1.41 (1.12, 1.76) **	1.40 (1.12, 1.76) **
1991–1993	3256	2464	75.7	2.40 (1.91, 3.01) ***	2.41 (1.91, 3.03) ***
1994–1996	5300	4202	79.3	2.95 (2.36, 3.68) ***	2.93 (2.34, 3.67) ***
***Parental country of birth***					
Two Norwegian-born parents	12,726	9276	72.9	1 (reference)	1 (reference)
Two immigrant parents	1174	765	65.2	0.70 (0.61, 0.79) ***	0.77 (0.67, 0.87) ***
One foreign-born parent and one Norwegian-born parent	1310	930	71.0	0.91 (0.80, 1.03)	0.90 (0.80, 1.03)
***Region of residence***					
East	7920	5626	71.0	1 (reference)	1 (reference)
South	762	512	67.2	0.84 (0.71, 0.98) *	0.81 (0.69, 0.96) *
West	3849	2847	74.0	1.16 (1.06, 1.26) ***	1.17 (1.07, 1.28) ***
Middle	1573	1184	75.3	1.24 (1.10, 1.41) ***	1.23 (1.08, 1.40) **
North	1097	797	72.7	1.08 (0.94, 1.25)	1.05 (0.91, 1.22)
***Maternal education***					
None/Primary only/Lower secondary	1889	1337	70.8	1 (reference)	--
Upper secondary/Post-secondary non-tertiary	4894	3631	74.2	1.19 (1.05, 1.34) **	--
Undergraduate	6182	4519	73.1	1.12 (1.00, 1.26) *	--
Postgraduate	1738	1197	68.9	0.91 (0.79, 1.05)	--
***Maternal occupation***					
Managers/Professionals/Technicians	7582	5558	73.3	1 (reference)	--
Others ^#^	3624	2675	73.8	1.03 (0.94, 1.12)	--
***Household annual income after tax (NOK)***					
<289,448	2572	1725	67.1	1 (reference)	1 (reference)
289,448–452,141	2255	1626	72.1	1.27 (1.12, 1.44) ***	1.06 (0.93, 1.22)
452,141–603,667	3603	2698	74.9	1.46 (1.31, 1.64) ***	1.12 (0.99, 1.27)
603,667–797,912	3608	2704	74.9	1.47 (1.31, 1.64) ***	1.13 (0.99, 1.28)
797,912–977,605	1609	1156	71.8	1.25 (1.09, 1.44) **	0.99 (0.85, 1.15)
977,605–	1561	1061	68.0	1.04 (0.91, 1.19)	0.81 (0.69, 0.94) **

^§^ Estimates adjusted for all variables in the tables (N = 15,211) except for maternal education level and maternal occupation which were not included due to a high proportion of missing values. ^#^ Others refers to clerical support workers, service and sales workers, skilled agricultural, forestry and fishery workers, craft and related trades workers, plant and machine operators and assemblers and elementary occupations. * *p* < 0.05; ** *p* < 0.01; *** *p* < 0.0001. Abbreviations: OR: odds ratios; CI: confidence interval.

**Table 3:** Sociodemographic characteristics of HPV vaccination initiation from November 2016 to June 2018 among women eligible for catch-up HPV vaccination in Norway.

Characteristics	No. Total	No. Initiated	% Initiated	Unadjusted HR (95% CI)	Adjusted HR (95% CI) ^§^
Overall	201,326	92,913	46.2	--	--
***Year of birth***					
1991–1992	71,065	30,733	43.2	1 (reference)	1 (reference)
1993–1994	66,436	30,948	46.6	1.10 (1.08, 1.12) ***	1.07 (1.05,1.08) ***
1995–1996	63,825	31,232	48.9	1.17 (1.15, 1.19) ***	1.11 (1.09,1.13) ***
***Parental country of birth***					
Two Norwegian-born parents	146,436	78,971	53.9	1 (reference)	1 (reference)
Two immigrant parents	35,141	7400	21.1	0.31 (0.31, 0.32) ***	0.40 (0.39, 0.41) ***
One foreign-born and one Norwegian-born parent	13,792	6221	45.1	0.77 (0.75, 0.79) ***	0.82 (0.80, 0.84) ***
***Region of residence***					
East	89,459	40,029	44.7	1 (reference)	1 (reference)
South	11,790	5535	46.9	1.06 (1.04, 1.10) ***	1.01 (0.98, 1.04)
West	41,618	22,177	53.3	1.27 (1.25, 1.29) ***	1.18 (1.16, 1.20) ***
Middle	27,506	14,591	53.0	1.28 (1.26, 1.31) ***	1.18 (1.16, 1.20) ***
North	19,839	9748	49.1	1.13 (1.10, 1.15) ***	1.03 (1.01, 1.06) **
***Maternal education***					
None/Primary only/Lower secondary	44,432	17,961	40.4	1 (reference)	--
Upper secondary/Post-secondary non-tertiary	72,506	37,704	52.0	1.41 (1.39, 1.44) ***	--
Undergraduate	50,762	29,461	58.0	1.66 (1.63, 1.69) ***	--
Postgraduate	8410	4871	57.9	1.64 (1.59, 1.69) ***	--
***Maternal occupation***					
Managers/Professionals/Technicians	63,778	37,952	59.5	1 (reference)	--
Others ^#^	66,372	32,902	49.6	0.76 (0.75, 0.78) ***	--
***Household annual income after tax (NOK)***					
<289,448	28,594	8301	29.0	1 (reference)	1 (reference)
289,448–452,141	45,160	19,082	42.3	1.56 (1.52, 1.60) ***	1.25 (1.22, 1.29) ***
452,141–603,667	61,797	32,054	51.9	2.08 (2.03, 2.13) ***	1.48 (1.44, 1.52) ***
603,667–797,912	40,031	22,371	55.9	2.32 (2.26, 2.38) ***	1.60 (1.56, 1.64) ***
797,912–977,605	11,198	6272	56.0	2.31 (2.23, 2.38) ***	1.60 (1.54, 1.65) ***
977,605–	8237	4509	54.7	2.24 (2.16, 2.32) ***	1.60 (1.54, 1.66) ***

^§^ Estimates adjusted for all variables in the tables (*N* = 201,326) except for maternal education level and maternal occupation which were not included due to a high proportion of missing values. ^#^ Others refers to clerical support workers, service and sales workers, skilled agricultural, forestry and fishery workers, craft and related trades workers, plant and machine operators and assemblers and elementary occupations. ** *p* < 0.01; *** *p* < 0.0001. Abbreviations: HR: hazard ratios; CI: confidence interval.

**Table 4:** Sociodemographic characteristics of HPV vaccination completion among women who initiated catch-up HPV vaccination from November 2016 to June 2018 in Norway.

Characteristics	No. Initiated	No. Completed	% Completed	Unadjusted OR (95% CI)	Adjusted OR (95% CI) ^§^
Overall	63,440	46,301	73.0	--	--
***Year of birth***					
1991–1992	21,149	15,528	73.4	1 (reference)	1 (reference)
1993–1994	21,017	15,317	72.9	0.97 (0.93, 1.02)	0.97 (0.93, 1.01)
1995–1996	21,274	15,456	72.7	0.96 (0.92, 1.00) *	0.96 (0.92, 1.00) *
***Parental country of birth***					
Two Norwegian-born parents	54,670	40,185	73.5	1 (reference)	1 (reference)
Two immigrant parents	4653	3177	68.3	0.78 (0.73, 0.83) ***	0.79 (0.74, 0.85) ***
One foreign-born and one Norwegian-born parent	4072	2915	71.6	0.91 (0.85, 0.97) **	0.91 (0.85, 0.98) *
***Region of residence***					
East	27,181	19,988	73.5	1 (reference)	1 (reference)
South	3773	2595	68.8	0.79 (0.74, 0.85) ***	0.79 (0.73, 0.85) ***
West	15,335	11,196	73.0	0.97 (0.93, 1.02)	0.96 (0.92, 1.00) *
Middle	10,293	7350	71.4	0.90 (0.85, 0.95) ***	0.88 (0.84, 0.93) ***
North	6557	4974	75.9	1.13 (1.06, 1.20) ***	1.11 (1.04, 1.18) **
***Maternal education***					
None/Primary only/Lower secondary	12,052	8630	71.6	1 (reference)	--
Upper secondary/Post-secondary non-tertiary	25,881	19,189	74.1	1.14 (1.08, 1.19) ***	--
Undergraduate	20,582	15,057	73.2	1.08 (1.03, 1.14) **	--
Postgraduate	3324	2348	70.6	0.95 (0.88, 1.04)	--
***Maternal occupation***					
Managers/Professionals/Technicians	26,476	19,408	73.3	1 (reference)	--
Others ^#^	22,546	16,656	73.9	1.03 (0.99, 1.07)	--
***Household annual income after tax (NOK)***					
<289,448	5402	3811	70.5	1 (reference)	1 (reference)
289,448–452,141	12,793	9283	72.6	1.10 (1.03, 1.18) **	1.08 (1.00, 1.16) *
452,141–603,667	22,107	16,257	73.5	1.16 (1.09, 1.24) ***	1.12 (1.04, 1.20) **
603,667–797,912	15,702	11,558	73.6	1.16 (1.09, 1.25) ***	1.11 (1.03, 1.19) **
797,912–977,605	4294	3113	72.5	1.10 (1.01, 1.20) *	1.04 (0.95, 1.14)
977,605–	3097	2255	72.8	1.12 (1.01, 1.23) *	1.06 (0.96, 1.17)

^§^ Estimates adjusted for all variables in the tables (N = 63,440) except for maternal education level and maternal occupation, which were not included due to a high proportion of missing values. ^#^ Others refers to clerical support workers, service and sales workers, skilled agricultural, forestry and fishery workers, craft and related trades workers, plant and machine operators and assemblers and elementary occupations. * *p* < 0.05; ** *p* < 0.01; *** *p* < 0.0001. Abbreviations: OR: odds ratios; CI: confidence interval.

## Data Availability

All data can be accessed by appropriate application to the public registries used in this study.
